# Design and fabrication of alginate hydrogel nanohybrid as a promising cancer treatment

**DOI:** 10.22038/IJBMS.2024.74226.16127

**Published:** 2024

**Authors:** Fatemeh Sadat Ajayebi, Nahid Hassanzadeh Nemati, Alireza Hatamirad, Mahrad Ghazli, Neda Attaran

**Affiliations:** 1 Department of biomedical engineering, Tehran Science and Research Branch, Islamic Azad University, Tehran, Iran; 2 Department of Medical Nanotechnology, Applied Biophotonics Research Center, Tehran Science and Research Branch, Islamic Azad University, Tehran, Iran

**Keywords:** Alginate hydrogel, Cancer treatment, Gold colloid, Nanostructures, Skin neoplasms

## Abstract

**Objective(s):**

Basal cell carcinoma (BCC) is the most common form of skin cancer and the most frequently occurring form of all cancers, affecting sun-exposed areas like the face. Surgery is the main treatment, focusing on safe and minimally invasive methods for better outcomes. Technology has enabled the development of artificial skin substitutes for tissue repair. Tissue engineering uses scaffolds to create functional replacements. This project aims to create an alginate-based hydrogel with PEG-coated gold nanoparticles.

**Materials and Methods:**

The project extensively explored the modification of alginate hydrogels with PEG-coated gold nanoparticles, involving the synthesis of gold nanoparticles, their integration with the polymer, and the subsequent preparation of the concentrated hybrid hydrogel. Utilizing various physicochemical techniques, such as UV-visible spectroscopy, transmission electron microscopy, dynamic light scattering, zeta potential analysis, scanning electron microscopy, and Fourier transform infrared spectroscopy, the fabrication process was optimized and characterized.

**Results:**

The successful synthesis of the hybrid biomaterial was achieved through robust and highly reproducible methods. The MTT assay results offered valuable insights into the biocompatibility and safety of the PEG-coated gold nanoparticle-loaded alginate-based films. The incorporation of PEG-coated gold nanoparticles allowed for potential drug loading on the nanoparticle surface and, consequently, within the hydrogel. Cellular assays were conducted to assess the potential applications of this novel biomaterial.

**Conclusion:**

The addition of polyethylene glycol made it possible to load different drugs onto the gold nanoparticles and also within the hydrogel. This makes it a promising choice for potential uses in tissue engineering.

## Introduction

In recent years, skin cancer has been identified as the most common type of cancer in developed and developing countries and is divided into two main groups: melanoma and non-melanoma. Basal cell carcinoma (BCC) and squamous cell carcinoma (SCC) are also classified as non-melanoma skin cancer (NMSC) (1).

BCC is the most common malignant tumor in humans, usually slow-growing and developing in sun-exposed areas of the body, especially on the face. Various treatment methods have been used for BCC, but surgery is the most common treatment for it. Surgical removal of BCC causes a defect in the skin and soft tissue that requires reconstruction. Skin grafting may also be used, but a major concern with skin grafts is color mismatch between the donor and recipient site. In planning for facial reconstruction, efforts should include the safest and least invasive methods to achieve optimal functional and aesthetic results. Recently, various artificial skin substitutes have been made using advanced technology for wound repair and improvement (2). Treating large acute or chronic wounds remains challenging due to the lack of effective methods for accelerating wound healing (3).

Wound healing is a complex biological and molecular event that involves various types of cells, including macrophages, fibroblasts, keratinocytes, and endothelial cells. In this process, fibroblasts play an important role in guiding angiogenesis and supporting the migration and proliferation of other cells by depositing extracellular matrix (ECM). The basis of wound healing is the cells that ultimately transform into scar tissue (4). Tissue engineering is a combined approach of engineering and biomedical sciences that has demonstrated a unique feature to create a biological substitute with structural and functional properties for tissue repair using scaffolds (5,6).

Hydrogels can stop bleeding, prevent bacterial infections, enhance collagen formation, maintain a moist environment, and fill irregular wounds. At their simplest form, hydrogels are water-loving cross-linked polymer networks that can absorb aqueous solutions up to a thousand times their dry weight and create gel-like conditions that simulate the niche of stem cells. Their high water content and permeability, as well as their adjustable viscoelasticity and structural similarity to the extracellular matrix, make hydrogels inherently suitable for biological applications. These key properties make them attractive for biomedical uses, which began with Wichterle and Lim in 1960 and have since continued as platforms for drug delivery and tissue engineering. Because of their structure and mechanical properties similar to native tissue extracellular matrix, high interaction with cells, and sufficient biochemical signaling to support cellular attachment, proliferation, and differentiation, hydrogels are some of the essential categories of biomaterials for scaffold fabrication. Hydrogels with injectability features are the latest research in this field. In addition, they are prepared without side effects and are simple with low toxicity. The stability of hydrogels is a prominent advantage that can be achieved by various methods of chemical and physical cross-linking to increase material adaptability and performance. The chemical integration of molecules that can guide cell activity or be released with time control can also be facilitated by advances in chemistry. Additionally, hydrogel networks can be used to encapsulate carriers such as nanostructures and nanoparticles for storage purposes (7, 9).

Nanoparticles are at the forefront of nanotechnology and have numerous potential applications (10-17). Hydrogel composites composed of metallic nanoparticles are produced and used for tissue engineering and drug delivery purposes. Metallic nanoparticles enhance properties such as strength, thermal stability, higher durability, and cellular compatibility for hydrogels. Gold nanoparticles play a role in cancer diagnosis and treatment, including as a carrier for gene delivery, biological detection of pathogens, and hyperthermia therapy for tumor destruction. Additionally, due to physical and chemical properties such as high conductivity, high catalytic activity at low temperatures, biocompatibility, localized surface plasmon resonance enhancement, surface performance, low toxicity, and antimicrobial activity, they have been studied extensively (7, 12). The higher antimicrobial properties of nanoparticles are due to their ability to penetrate more easily into the cell membranes and walls of pathogens than conventional antifungal and antibacterial drugs.

Many metallic nanoparticles are currently widely available commercially and are used mostly in hospitals for wound dressing. Some of these metal ions have a broad spectrum of antimicrobial activity with multiple cellular targets. This spectrum of action is usually due to the unique properties of the metal, which enable its interaction with relevant functional groups in biological molecules. The selectivity and specificity of the metal binding are strongly associated with its antimicrobial effects. Depending on the metal properties, different metals target the substitute functional groups in metabolites, proteins, nucleic acids, lipids, and carbohydrates, as each of these components can trigger harmful cascade reactions in the cell. We can attribute the impact of the previous cascade to disruption of membrane integrity, direct and indirect changes in membrane potential and transport across the membrane, malfunctioning of proteins and metabolism stimulating denaturation, electron transfer chain disruption, DNA damage and structural alteration, inhibition of DNA replication and repair, and degradation of carbohydrates.

The generation or stimulation of reactive oxygen species (ROS) is another mechanism by which metals interfere with harmful effects. Some redox-active metals have the capacity to donate or accept electrons from different atoms. Redox-active metals catalyze photon-driven reactions that generate active oxygen species. The inhibition of proteins or other compounds involved in redox balance or electron transfer chains may also indirectly lead to an increase in ROS levels. Direct or indirect stimulation of ROS in cells can lead to damage to proteins, nucleic acids, and membranes.

The antimicrobial spectrum of gold nanoparticles can be attributed to two characteristics, the release of metal ions from nanoparticles and the inherent properties of the nanoparticle itself (18). Concurrently, the type of metal, and the specific nanoparticle properties, collaborate with the antimicrobial spectrum observed. For example, smaller nanoparticles with a higher surface-to-volume ratio are usually more dangerous for bacteria, most likely due to their ability to pass through the cell membrane. The rate of metal decay also plays an important role in the degree of toxicity as rapidly dissolving nanoparticles are often associated with increased harmful effects. Overall, it appears that metal ions are the main source of toxicity for microorganisms, and nanoparticles act as sophisticated vehicles that accumulate the release of metal ions at the cell surface. Gold nanoparticles with epigallocatechin gallate and α-lipoic acid hydrogel have been proven very effective anti-inflammatories and potent anti-oxidants in wound healing. Gold nanoparticles with collagen exhibit regenerative properties that depend on the amount of wound repair properties, demonstrating skin wound healing. Many studies have shown that hydrocolloid membranes coated with gold nanoparticles considerably reduce the healing process. Various investigations into the properties of AuNP have demonstrated their oxidative and antimicrobial properties, which prove a powerful aspect in the regeneration of damaged collagen fibers and strengthening the wound healing process. Gold nanoparticles accelerate the wound healing process through their anti-inflammatory and anti-angiogenic properties by increasing the spread of vascular endothelial growth factor (VEGF), IL-12, IL-8, and TNF-α (19, 20).

The choice of polymer for stabilizing gold nanoparticles is based on its capacity to create cavities in the polymer network during the swollen state, acting as a site for nucleation and growth of nanoparticles. For wound applications, the dressing may be used in the form of a hydrogel, which can be produced from natural, synthetic, or semi-synthetic polymers. Those with a natural origin have the highest usage due to their biodegradability and biocompatibility with the human body. Composites containing gold nanoparticles with naturally derived materials such as alginate, chitosan, heparin, keratin, proteoglycans, collagen, gelatin, fibrin, and silk fibroin are suitable for accelerating fibroblast migration, angiogenesis, granulation tissue formation, and reducing pro-inflammatory cytokines. If formulated with gold nanoparticles, an aqueous environment is required to facilitate the release of nanoparticles from the polymeric network and maintain hydration of the wound for better healing. The unique and tunable properties of nanocomposite hydrogels have been utilized for antimicrobial and wound-healing applications (21, 22).

The modified alginate-based hydrogel with PEG-coated gold nanoparticles offers a range of advantages and applications in treating BCC and wound healing, including 1) Cancer Treatment (23); i) Inhibiting DNA self-repair in breast cancer treatment. ii) Promoting blood circulation and enhancing brachytherapy efficacy in breast cancer treatment. 2) Wound Infection Prevention (24); i) Minimizing bacterial infections at the wound site. ii) Preventing wound infection by absorbing excess wound fluid and maintaining a moist environment. 3) Wound Healing (25-29); i) Rapidly releasing dye molecules under UV light, potentially aiding in acute wound healing. ii) Enhancing skin re-epithelization and collagen deposition for wound healing. iii) Promoting efficient and rapid wound healing for skin wounds. iv) Accelerating diabetic wound healing by reducing bacterial-induced chronic inflammation and promoting angiogenesis. v) Enhancing the spreading and proliferation of fibroblasts and promoting osteogenic differentiation of mesenchymal stem cells in 3D cell culture. 4) Antibacterial Activity (30); i) Providing potent antibacterial ability, accelerating wound healing, and effectively absorbing blood and tissue exudates. 5) Material Enhancement (31-33); i) Enhancing wound dressing material by increasing compatibility, strength, and antibacterial resistance. ii) Enhancing *in vivo* durability and physical properties for cell transplantation applications. iii) Assisting in cell adhesion and cell culture applications in the treatment of BCC. 6) Scar Treatment (34); i) Treating scars and reducing scar formation, benefiting wound healing applications.

According to the statements made, this article discusses the synthesis and fabrication of an alginate hydrogel composite incorporating gold nanoparticles, in order to potentially be used as an effective composite in the production of cellular dressings and wound dressings for use in the treatment of skin diseases, including skin cancer.

## Materials and Methods

***Materials and equipment***

The reagents utilized in this study were sodium alginate, polyethylene glycol (H(OCH_2_CH_2_)_n_OH, average mol wt. 2,000), hydrogen tetrachloroaurate (III) trihydrate (HAuCl_4_·3H_2_O, 99.5% purity), and sodium citrate dihydrate (C_6_H_5_Na_3_O_7_·2H_2_O), which were purchased from Merck, Germany and Fluka, Switzerland. The MCF7 breast cancer cell line was acquired from the Pasteur Institute of Iran and cultured in RPMI-1640 medium procured from GIBCO (Invitrogen, Germany). Additional reagents including trypsin-ethylene diamine tetraacetic acid (EDTA), dimethyl sulfoxide (DMSO), 3-(4,5-dimethylthiazol-2-yl)-2,5-diphenyltetrazolium bromide (MTT), and penicillin-streptomycin solution were also obtained from Sigma-Aldrich Corp. 

The optical absorption spectrum of varying sample concentrations was evaluated using a visible-ultraviolet light spectrophotometer (HACH, USA, model DR6000) at wavelengths ranging from 200 to 800 nm. Transmission electron microscopy (TEM) with a Zeiss EM 900 from Germany was employed to characterize nanoparticle morphology and size. Scanning electron microscopy (SEM), dynamic light scattering (DLS) through a ZS-90 device from Malvern Instruments, UK, and zeta potential measurement via a measuring device (Malvern Zetasizer Nano ZS-90) from Malvern Instruments, UK were utilized to determine particle size distribution, hydrodynamic diameter, and electric charge of particles, respectively. FTIR spectra of the samples were obtained using a Thermo Nicolet Avatar 370 FTIR spectrometer from Thermo Fisher Scientific, USA. A vortex mixer (VM-10, WITEG Labortechnik) is used for mixing solutions inside small vials and tubes. The plastic ring on the device that holds the vial or tube undergoes vibration and rotary and orbital movement, causing the sample to mix. The MTT cell viability assay was used test to assess the toxicity of compounds on cells. It distinguishes between living and dead cells by measuring the metabolic activity of cells.

***Methods***

*Synthesis of polyethylene glycol-coated gold nanoparticles*

Gold nanoparticles were synthesized using the citrate reduction method, as reported previously (35). Specifically, 0.01 g of HAuCl_4_ gold salt was added to 5 ml of deionized water and then added to 90 ml of water in a round bottom flask on a magnetic stirrer. The solution was boiled and sodium citrate rapidly added, resulting in a color change from pale yellow to purple and eventually to dark red. After achieving red color, it was refluxed for another 15 min and allowed to cool at room temperature.

To stabilize the synthesized gold nanoparticles, 0.1 ml of a 3% solution by weight of polyethylene glycol (PEG) was added to 10 ml of an aqueous solution of gold nanoparticles and placed on a magnetic stirrer for 4 hr at room temperature. PEG is used as a coating agent on the surface of gold nanoparticles to prevent their settling and stabilize the distribution of nanoparticles, making them more effective in targeting cells (36).

*Preparation of alginate films loaded with gold nanoparticles*

To create alginate hydrogels, a solution of 4 wt.% alginic acid sodium salt was drop cast. Various samples were prepared to compare the impact of incorporating gold nanoparticles into the hydrogel matrix. These samples included alginate films without gold nanoparticles, alginate films with non-coated gold nanoparticles, and alginate films with coated gold nanoparticles. Different amounts of coated and non-coated gold nanoparticles were used in the films.

**Figure 1:**
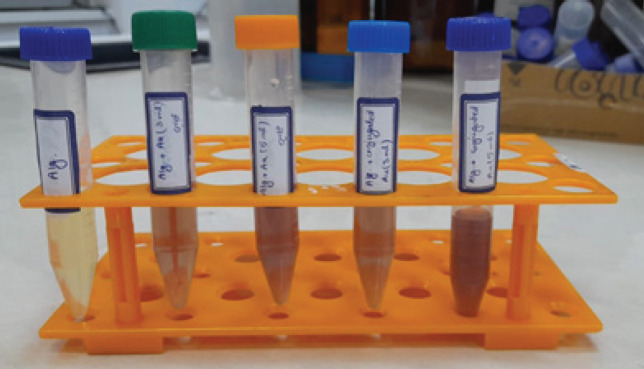
Image of a 4% w/v sodium alginate solution with varying percentages of gold nanoparticles

Various samples preparation was done as follows (Figure 1):

1. 0.2 gr of alginate acid salt was dissolved in 5 milliliters of deionized water to obtain a 4% weight solution of sodium alginate. 

2. 0.2 gr of alginate acid salt was mixed with 2 milliliters of water and 3 milliliters of gold nanoparticle solution (alginate with 60% gold solution). 

3. 0.2 gr of alginate acid salt was mixed with 5 milliliters of gold nanoparticle solution (alginate with 100% gold solution). 

4. 0.2 gr of alginate acid salt, 2 milliliters of deionized water, and 3 milliliters of polyethylene glycol-coated gold nanoparticle solution (alginate with 60% polyethylene glycol-coated gold solution). 

5. 0.2 gr of alginate acid salt was mixed with 5 milliliters of polyethylene glycol-coated gold nanoparticle solution (alginate with 100% polyethylene glycol-coated gold solution).

**Figure 2:**
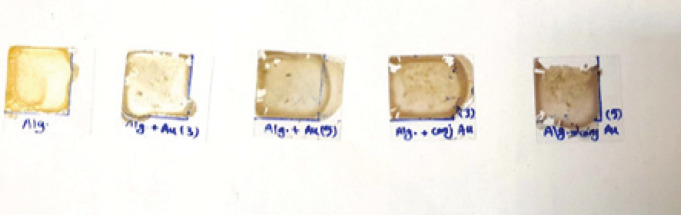
Hydrogel film of different alginate samples

The film preparation process involved drop-casting a specific mass (0.25 g) of the solution onto a microscope slide, as depicted in Figure 2. The film area was controlled to ensure comparable thicknesses (1.5x1.5 cm). Care was taken to avoid bubble formation by gently casting the solution onto the substrate. The sample was then allowed to rest for 30 min to achieve a uniform distribution (37).

*Preparation of crosslinked alginate gel with gold nanoparticles through calcium chloride*

**Figure 3:**
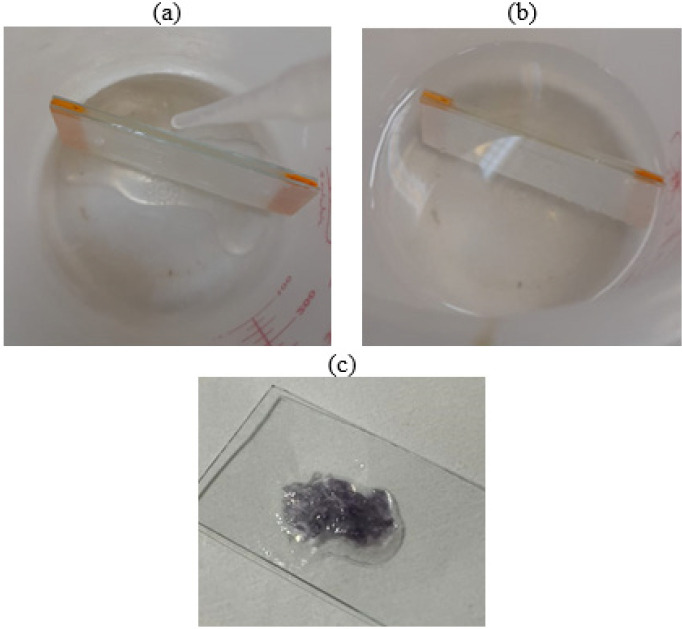
a) and b) Preparation stages of alginate gel, c) Final alginate gel

Crosslinking of polymer chains to obtain a specific three-dimensional structure of hydrogels was performed by adding 0.1 M calcium chloride solution. In Figure 3, two slides were placed on top of each other and a small foam rectangular strip was placed between them at two opposite sides to create a distance. Then, a strip of parafilm was wrapped around the slides in a way that only one side remained open and the other sides were covered by parafilm. Alginate solution was poured dropwise between the two slides through the open distance. The slides were then immersed in the calcium chloride solution for 2 hr to form crosslinking. The samples were placed in the calcium chloride bath, ensuring that they were completely covered, and Ca^+2^ was completely released during 2 hr. After this period, the slides were removed, and the gel was gently separated from the slides by carefully opening the parafilm around it.

## Results

***Polyethylene glycol-coated gold nanoparticles characterization***

Gold nanoparticles are synthesized by the Turkevich method, a well-known method developed by Turkevich in 1951. A homogeneous gold suspension with well-dispersed particles is obtained by chemically reducing a metal salt. Tetrachloroauric acid (HAuCl_4_) is reduced in an aqueous solution using sodium citrate (Na_3_Ct). Sodium citrate acts as a reducing and coating agent, helping to stabilize the gold particles. The Turkevich method is widely used due to its simplicity and the ability to control the final particle size. It is a mechanism of particle growth through nucleation, in which the number of nanoparticles is defined in the initial synthesis stage and remains constant during the reaction. Therefore, the particle size depends on the ratio of citrate to gold. 

The goal of surface modification of gold nanoparticles with polymers is to increase their stability and prevent particle aggregation, as well as improve their binding to biomolecules and drugs. 

The unique optical properties of gold nanoparticles allow for their characterization using UV-Vis spectroscopy, a straightforward technique. Unlike bulk gold, colloidal gold solutions at the nanoscale exhibit vibrant colors such as red or purple. This color variation arises from the interaction between electrons on the surface of the nanoparticles and specific wavelengths of light. This interaction leads to a collective oscillation of electrons in the conduction band called surface plasmon resonance (SPR). The size, shape, and aggregation state of gold nanoparticles influence the extinction spectrum produced by SPR (38). In aqueous solutions, gold nanoparticles display a broad absorption band around 520 nanometers in the visible region. However, SPR is not observed in gold nanoparticles smaller than 2 nanometers in diameter or bulk gold.

**Figure 4:**
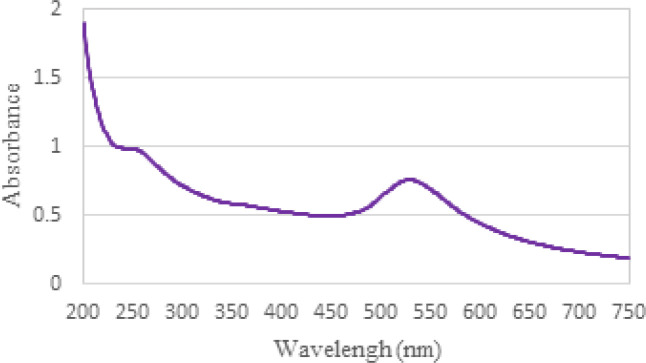
UV-visible absorption spectrum of gold nanoparticles solution

In the case of the synthesized gold nanoparticle solution, the peak of the spectrum occurs at a wavelength of 528.0 nanometers (Figure 4). The intensity of this peak is attributed to the enhancement of plasmon resonance in gold nanoparticles.

**Figure 5:**
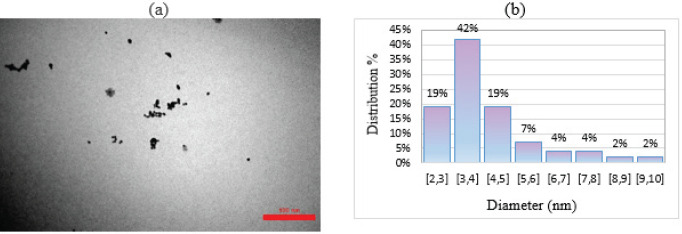
a) TEM image of gold nanoparticles, b) Histogram plot of particle size distribution in gold nanoparticles solution

Transmission electron microscopy (TEM) is the most common method for obtaining images to calculate the size distribution of NPs due to the easy sample preparation. First, TEM images were taken and then scanning electron microscopy (SEM) was used for the same purpose. The principle of operation of these two microscopes is the same. In this way, the bright field images are analyzed with electrons passing through the transparent sample. The difference between them is that in SEM, the electron beam scans the sample, while in TEM, the beam is static. In Figure 5a, gold nanoparticles are clearly visible and dispersed in the solution. The average diameter of the nanoparticles was determined by analyzing the TEM images. The Digimizer software was used to process the images and draw the particle size distribution histogram. The calculated average diameter is 5.202 nanometers (Figure 5b). As can be seen, the spherical particles are single and dispersed following the synthetic method previously described.

The solution of gold nanoparticles was subjected to measurement of dynamic light scattering diffraction (DLS). Based on the graphs and analyzed data, the hydrodynamic diameter of the gold nanoparticles is 39.0 nanometers with a scatter index of 0.870. The solution of gold nanoparticles was tested for zeta potential. The measured zeta potential of the gold nanoparticles is -10.9.

**Figure 6:**
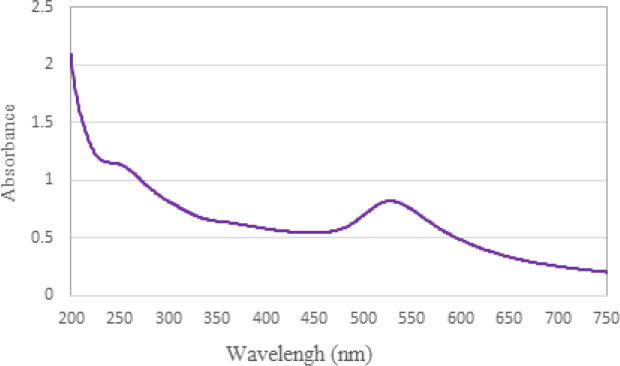
UV-visible absorption spectrum of PEG-coated gold nanoparticle solution

The properties of the polymer-coated gold nanoparticles were studied. The gold nanoparticles were coated with polyethylene glycol (PEG) after synthesis. In the gold nanoparticle solution, the peak on the spectrum at a wavelength of 530.0 nanometers is due to the surface plasmon resonance of the nanoparticles. The presence of PEG was confirmed through UV-Vis spectroscopy (Figure 6). The change in the maximum peak intensity, compared to pure gold nanoparticles, indicates conjugation. PEG creates a shell around the gold nanoparticles, which alters the nanoparticle environment and causes a shift in the surface plasmon band (SPB) redshift due to different light reflections.

**Figure 7:**
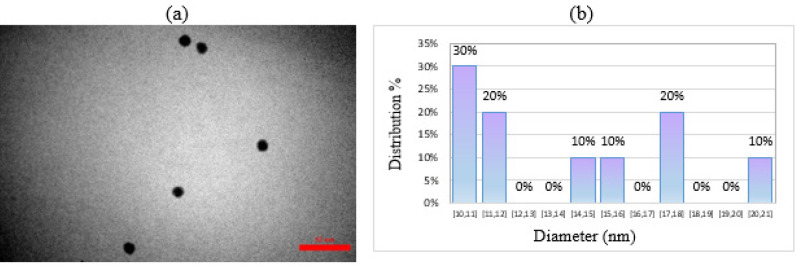
a) TEM image of PEG-coated gold nanoparticles, b) Histogram plot of particle size distribution in PEG-coated gold nanoparticle solution

TEM: Transmission electron microscopy; PEG: Polyethylene glycol

The gold nanoparticle solution coated with polyethylene glycol was observed using transmission electron microscopy. In Figure 7a, the PEG-coated nanoparticles are seen as individual and scattered particles in the solution. The Digimizer software was used to process images and draw histograms of particle size distribution. The calculated average diameter is 10.823 nanometers (Figure 7b). The spherical single-scattered particles were obtained through a previously described synthesis method.

The polyethylene glycol-coated gold nanoparticle solution was also tested for light scattering diffraction. Based on the graphs and analysis data, the hydrodynamic diameter of the PEG-coated nanoparticles is 90.1 nanometers with a scatter index of 0.390. In conclusion, it can be inferred that the hydrodynamic diameter of the polymer-coated gold nanoparticles is larger than that of the uncoated ones. The polyethylene glycol-coated gold nanoparticle solution was tested for zeta potential. The calculated zeta potential for the PEG-coated gold nanoparticles is -4.5. In all of these analyses, the electrode voltage was 3.3 volts for both samples. Therefore, it is evident that the zeta potential has increased for the polymer-coated gold nanoparticles. The lower the particle potential, the greater their tendency to settle down and aggregate. Consequently, the polymer-coated gold nanoparticle solution is more stable than the pure gold nanoparticles.

***Characterization of alginate-based films loaded with PEG-coated gold nanoparticles***

Loading alginate with PEG-coated gold nanoparticles can add new properties. For example, increasing the stability of gold nanoparticles can increase the time it takes for active ingredients to be loaded into alginate, thereby increasing its useful life. Additionally, PEG-coated gold nanoparticles can be used for medical imaging because they have the ability to be labeled. Furthermore, loading alginate with PEG-coated gold nanoparticles can increase interactions with biomolecules, thereby improving drug development and treatment.

The alginate hydrogels were prepared by dripping a drop of 4% (w/v) sodium alginate salt solution. The alginate hydrogels are transparent, while the loaded hydrogels show a bright pink color due to the presence of gold nanoparticles. The color is more intense for higher concentrations of gold nanoparticles. The color homogeneity is the first indication of uniform nanoparticle distribution in the hydrogel.

**Figure 8:**
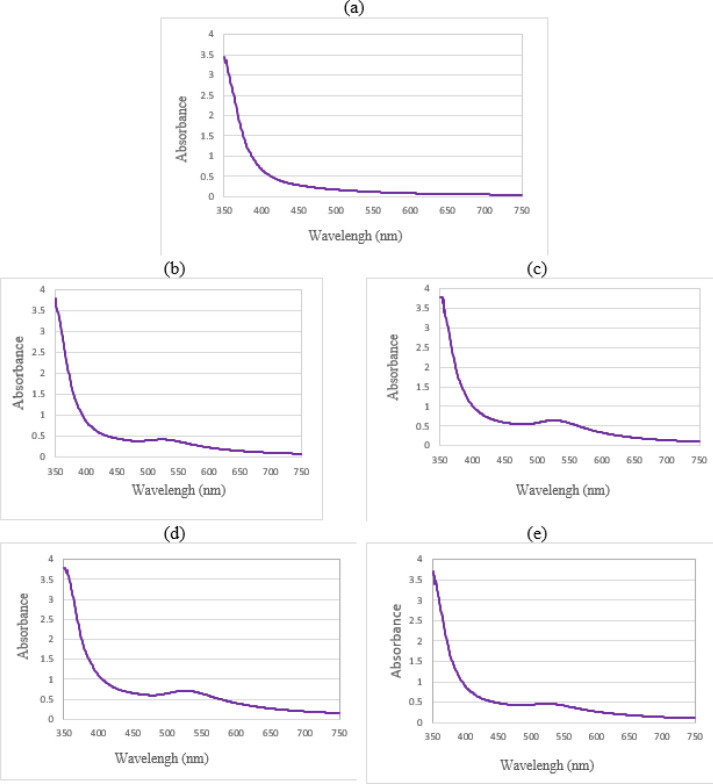
UV-visible absorption spectrum of a) unloaded alginate film, b) alginate with 60% non-coated gold solution, c) alginate with 100% non-coated gold solution, d) alginate with 60% PEG-coated gold solution, and e) alginate with 100% PEG-coated gold solution

The UV-vis spectrum obtained for the prepared hydrogels is presented in Figure 8. As expected, no absorption band is visible for the unloaded alginate film. The SPB feature from gold nanoparticles appears for the other samples but is shifted toward longer wavelengths compared to the PEG-coated gold nanoparticles solution in Figure 6, due to interaction with the alginate network. Thus, the presence of gold nanoparticles in the hydrogel matrix has been confirmed. On the other hand, the intensity of the peak is affected by the concentration of gold nanoparticles. As expected, the higher the concentration of nanoparticles, the higher the peak intensity. This trend is observed for films containing coated or non-coated gold nanoparticles (alginate with 60% non-coated gold solution, alginate with 100% non-coated gold solution, alginate with 60% PEG-coated gold solution, and alginate with 100% PEG-coated gold solution). The absorbed wavelength in the alginate solution with 60% gold nanoparticles is 522.0 nanometers, while it is 525.0 nanometers for alginate with 100% gold nanoparticles, and for alginate with 60% PEG-coated gold solution and 100% PEG-coated gold solution, it is 523.0 and 525.0 nanometers, respectively. Although the absorbed wavelength increased slightly with an increase in gold percentage in the solution, the presence of PEG had no effect on the absorption intensity.

**Figure 9:**
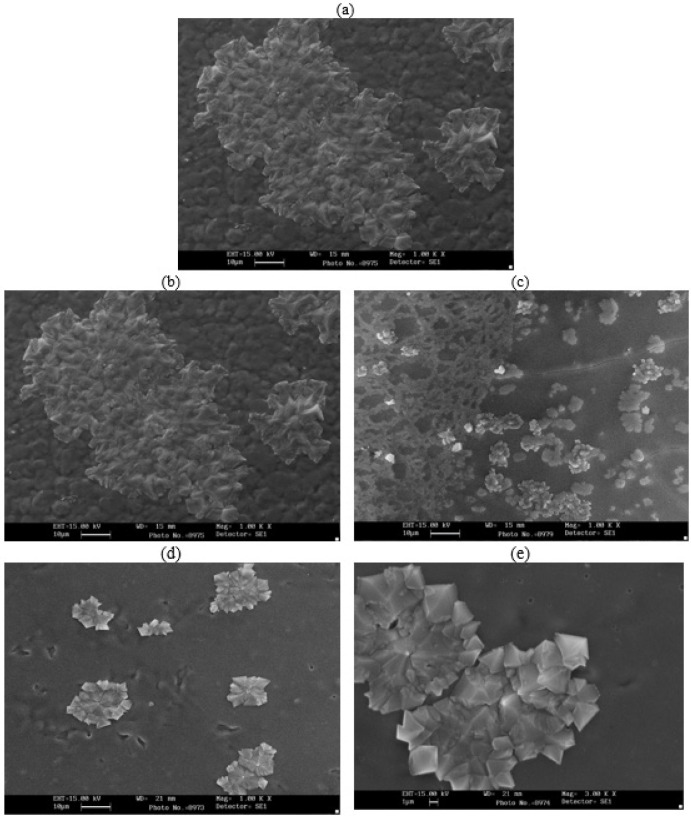
SEM image of a) pure alginate film, b) alginate with 60% non-coated gold solution, c) alginate with 100% non-coated gold solution, d) alginate with 60% PEG-coated gold solution, and e) alginate with 100% PEG-coated gold solution. (Scale: 10 micrometers)

To analyze the distribution of gold nanoparticles in alginate films, SEM images (Figure 9a) were used for the prepared films. As predicted, no gold nanoparticles are present in the control alginate film. However, gold nanoparticles can be seen in all loaded samples as brighter spots than the background due to the higher atomic number of gold. The non-coated gold nanoparticles are more sparsely distributed in the hydrogel matrix compared to the active particles. The active particles likely have a higher accumulation in certain regions due to chain interactions.

In SEM images, Figure 9a shows the pure alginate film. The background displays a uniformity in which non-spherical alginate particles with angular structures are dispersed. In Figures 9b and 9c, we can observe voids in the background that were not present in the pure alginate film. However, with the incorporation of coated gold nanoparticles (Figures 9d and 9e), we see a decrease in the voids of the background. Additionally, it appears that the particles have better solubility than the pure gold nanoparticle solution because large alginate particles are not visible in the image; instead, small spherical and non-angular particles are dispersed in the background. Overall, the solution film with coated gold nanoparticles seems to be more homogeneous and contains smaller particles. In the above images, cracks in the background can be seen due to the formation of cracks in the film.

The FT-IR spectrum of alginate film typically exhibits characteristic peaks corresponding to various functional groups present in the alginate molecule. The exact positions and intensities of these peaks may vary depending on the source of alginate and its composition (39). The peak appears around 3424 cm^-1^ and corresponds to the stretching vibration of hydroxyl (OH) groups present in the alginate molecule. The peaks can be observed in the range of 2900–3000 cm^-1^ and are related to the stretching vibration of carbon-hydrogen (C-H) bonds. The peak around 1630 cm^-1^ corresponds to the stretching vibration of the carbonyl (C=O) groups, which are typically present in the uronic acid residues of alginate. The peak appears around 1031 cm^-1^ representing the stretching vibration of C-O bonds, present in the alginate molecule. Alginate contains carboxylic acid groups, and their vibrations produce characteristic peaks in the region of 1550 cm^-1^.

**Figure 10:**
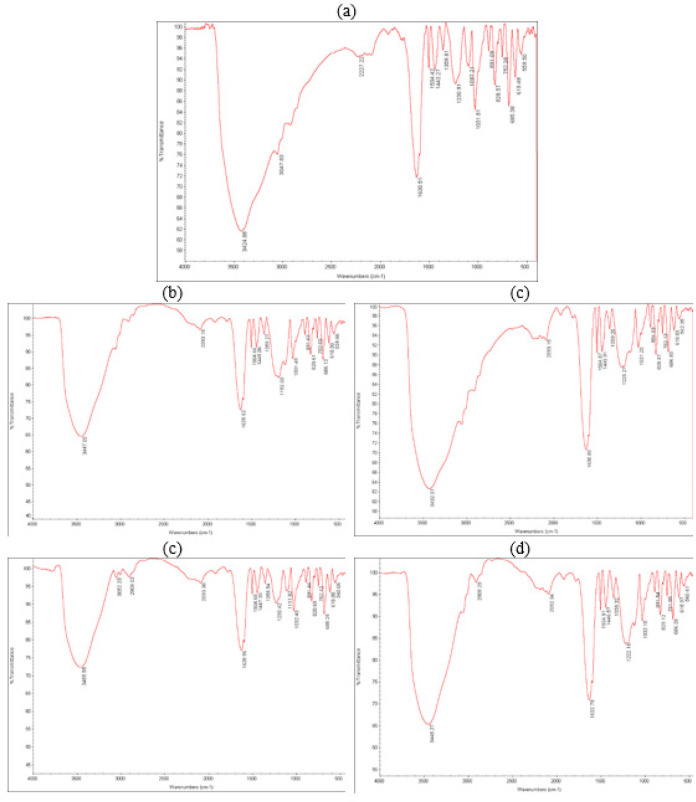
FT-IR spectrum of a) pure alginate film, b) alginate with 60% non-coated gold solution, c) alginate with 100% non-coated gold solution, d) alginate with 60% PEG-coated gold solution, and e) alginate with 100% PEG-coated gold solution.

It’s important to note that the specific peak positions and intensities of the FT-IR spectrum of alginate vary based on the presence of metal particles (non-coated and coated gold nanoparticles) (Figure 10 b-e).

***Toxicity of alginate-based films loaded with PEG-coated gold nanoparticles***

The toxicity of alginate-based films loaded with PEG-coated gold nanoparticles was evaluated in this study. The films were prepared using alginate as the main matrix material, and PEG-coated gold nanoparticles were incorporated into the film structure. The purpose of adding PEG-coated gold nanoparticles to the alginate film may have been to enhance certain properties or enable specific functionalities for potential applications. To assess the toxicity, various biological or cellular assays might have been performed. Common methods for assessing cytotoxicity include the MTT assay, cell viability tests, or studies to examine cellular responses and potential adverse effects caused by exposure to the films. The results of the toxicity evaluation would have provided insights into the biocompatibility and safety of the alginate-based films loaded with PEG-coated gold nanoparticles. This information is essential for potential biomedical or pharmaceutical applications, as it helps to determine whether the materials are suitable for use in contact with living cells or organisms without causing harm or adverse reactions. The study’s findings would have implications for the development and design of novel nanocomposite materials for various applications in medicine, drug delivery, or other fields.

**Figure 11:**
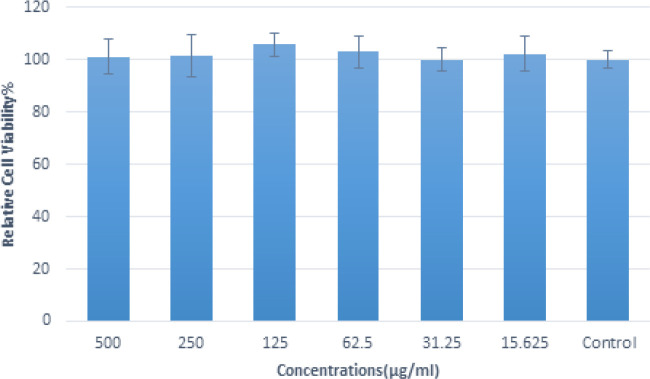
Percentage of viable melanoma skin cancer cells that have been incubated with different concentrations of alginate-based films loaded with PEG-coated gold nanoparticles

The toxicity of alginate-based films loaded with PEG-coated gold nanoparticles was tested using the MTT assay. The MTT assay is a commonly used colorimetric assay for assessing cell viability and determining the cytotoxicity of substances or materials. In the MTT assay, the A375 skin melanoma cell line is treated with the alginate-based films loaded with PEG-coated gold nanoparticles under investigation (Figure 11). Approximately 1×10^4^ cells are cultured in each well by counting them using trypan blue. After culturing the cells, the 96-well plate is placed in an incubator at 37 °C for 24 hr until 60% of the wells are filled. Different dilutions are required for treating the cells, and all dilutions must be prepared in sterile microtubes. The supernatant of each well is emptied using a sampler, and then 100 µl of each dilution (at concentrations of 500, 250, 125, 62.5, 31.25, and 15.625 µg/ml) is added to the corresponding wells. A pouring pattern is drawn, and eight wells are allocated for each dilution. After 24 hr, the medium is removed, and 100 µl of MTT solution (at a concentration of 0.5 mg/ml) is added to the plates in the dark, followed by placement in the incubator for 4 hr. Subsequently, the top medium of the wells is removed using a sampler, and 100 µl of DMSO is added to the wells. The plate is then placed on a shaker for 20 min (at this stage, the container should be covered to prevent exposure to light). Finally, the intensity of the resulting color is measured using a microplate reader (DNM-9602G) at a wavelength of 570 nm. The absorbance values obtained from samples treated with the hybrid biomaterial are compared to the control sample (untreated cells). The extent of cell viability is then calculated by comparing the absorbance values between the treated and control samples.

It was revealed that across a wide range of concentrations (15 to 500 micrograms/ml), the alginate-based films loaded with PEG-coated gold nanoparticles displayed a survival rate of approximately 100%. These findings provide strong evidence supporting the complete safety and compatibility of this combination.

TEM: Transmission electron microscopy

PEG: Polyethylene glycol

PEG: Polyethylene glycol

PEG: Polyethylene glycol

PEG: Polyethylene glycol

PEG: Polyethylene glycol

## Discussion

Gold nanoparticles are synthesized using the Turkevich method, involving the reduction of HAuCl4 with sodium citrate. This method is simple and allows control over particle size. Gold nanoparticles possess intriguing optical properties stemming from surface plasmon resonance (SPR), which gives rise to their vivid colors. The characteristic peak observed at 528.0 nm in the spectrum of the synthesized solution signifies an intensified plasmon resonance phenomenon. This phenomenon occurs when light interacts with the collective oscillation of electrons on the surface of the gold nanoparticles, causing the absorption and scattering of specific wavelengths. The precise control of synthesis conditions, such as nanoparticle size and shape, allows for fine-tuning of the SPR properties, influencing the resulting optical behavior and color expression of gold nanoparticles. Transmission Electron Microscopy (TEM) is a widely employed technique for analyzing the size of nanoparticles. In this study, TEM images were utilized to visually observe the synthesized gold nanoparticles. The images revealed that the nanoparticles were clearly visible and well-dispersed, exhibiting an average diameter of 5.202 nm. Furthermore, the particles appeared to exhibit a spherical shape, which aligns with the specific synthetic method employed. In addition to TEM, DLS and zeta potential measurements were employed to further evaluate the gold nanoparticles. DLS analysis provided insight into the hydrodynamic diameter of the nanoparticles, indicating a diameter of 39.0 nm. This measurement takes into account the size of nanoparticles as well as their surrounding solvent molecules, providing information on their behavior in solution. Furthermore, the scatter index of the gold nanoparticles was determined to be 0.870. This parameter describes the extent of scattering observed from the nanoparticles, indicating their potential stability and uniformity in solution. Collectively, the combination of TEM, DLS, and zeta potential measurements allows for a comprehensive characterization of the gold nanoparticles synthesized in this study. These results provide valuable information about the size, morphology, and stability of the particles.

The surface modification of gold nanoparticles with a polymer has been shown to have significant benefits, such as improved stability and enhanced binding properties. In this study, the synthesized gold nanoparticles were coated with a PEG polymer, leading to observable changes in their properties. One notable change observed was a redshift in the surface plasmon band (SPB) of the PEG-coated gold nanoparticles, resulting in a shift to a wavelength of 530.0 nm. This shift indicates a modification in the nanoparticles’ optical properties, which can be attributed to the presence of the polymer layer. Transmission Electron Microscopy (TEM) analysis was performed to investigate the morphology and size of the PEG-coated gold nanoparticles. The TEM images revealed that the particles appeared as single-scattered spherical entities with an average diameter of 10.823 nm, consistent with the synthesis method employed. However, the PEG-coated gold nanoparticles exhibited a larger hydrodynamic diameter of 90.1 nm compared to the uncoated nanoparticles. The hydrodynamic diameter accounts for the size of the nanoparticles in the solution along with the polymer layer and any solvent molecules associated with them. This increase in size suggests that the PEG coating contributes to the overall size of the nanoparticles in a solution environment. To assess the stability of the PEG-coated nanoparticles, the scatter index was determined to be 0.390. This value indicates a lower degree of scattering compared to uncoated nanoparticles, suggesting an enhanced potential for stability in solution. The reduced scattering is attributed to the presence of the polymer layer, which may help prevent particle aggregation and improve their overall stability. Furthermore, the zeta potential of the PEG-coated nanoparticles was found to be -4.5, indicating an increase in stability and reduced propensity for particle aggregation. The negative zeta potential suggests that the nanoparticles possess a surface charge that helps repel mutually, minimizing the likelihood of agglomeration. Overall, the introduction of a polymer coating, specifically PEG, to the gold nanoparticles resulted in a solution with enhanced stability compared to pure gold nanoparticles. The coating not only caused changes in the optical properties but also increased the hydrodynamic diameter and improved the zeta potential. These findings support the notion that surface modification using polymers can significantly impact the behavior and performance of nanoparticles, offering potential advantages in various applications such as drug delivery, sensing, and biotechnology.

The loading of alginate with PEG-coated gold nanoparticles brings about several advantageous effects, including improved stability, extended useful life, enabling medical imaging, enhanced interactions with biomolecules, and support for drug development. These benefits make it a promising approach for various applications. Hydrogels prepared with sodium alginate salt solution appear transparent. However, when loaded with PEG-coated gold nanoparticles, the hydrogels exhibit a vibrant pink color, indicating the presence and uniform distribution of the nanoparticles. This visible color change confirms the successful loading of the gold nanoparticles into the alginate matrix. The UV-vis spectrum of the hydrogels provides further insight into their characteristics. Unloaded alginate lacks an absorption band in the measured range, demonstrating the absence of any significant absorbance. However, the spectrum of the loaded hydrogels reveals the appearance of the surface plasmon band (SPB) feature of the gold nanoparticles. In these samples, the SPB has shifted towards longer wavelengths, indicating an interaction between the gold nanoparticles and the alginate matrix. The intensity of the SPB peak increases with higher nanoparticle concentration, confirming the presence of gold nanoparticles within the hydrogel. The absorbed wavelengths vary depending on the specific composition of the hydrogels, highlighting the tunability of the system. Importantly, the presence of PEG coating on the gold nanoparticles does not affect the absorption intensity, suggesting that the protective coating does not hinder their optical properties. To analyze the distribution of gold nanoparticles within the alginate films at a microscopic level, scanning electron microscope (SEM) images are utilized. Loaded samples exhibit brighter spots corresponding to the presence of gold nanoparticles. Additionally, the active particles tend to accumulate in specific regions due to chain interactions within the alginate matrix. The introduction of coated gold nanoparticles into the alginate films also has a significant impact on their overall structure. Voids in the film decrease, and smaller particles disperse more uniformly, resulting in a more homogeneous appearance. The Fourier-transform infrared (FT-IR) spectrum of the alginate film provides valuable information about the functional groups present. The positions and intensities of characteristic peaks depend on the specific source and composition of the alginate. Furthermore, the presence of metal particles, both non-coated and coated gold nanoparticles, alters the peak positions and intensities in the spectrum, indicating interactions between the alginate and the nanoparticles. Therefore, loading alginate with PEG-coated gold nanoparticles offers a range of benefits, including improved stability and prolonged useful life. The presence of gold nanoparticles enables medical imaging, enhances interactions with biomolecules, and supports drug development. The characterization techniques employed, including UV-vis spectroscopy, SEM imaging, and FT-IR analysis, provide valuable insights into the behavior and distribution of the gold nanoparticles within the alginate matrix. These findings contribute to our understanding of the interaction between nanoparticles and polymers, facilitating the design and development of advanced nanocomposite materials for various biomedical applications.

The toxicity of alginate films loaded with PEG-coated gold nanoparticles was assessed using the MTT assay. This commonly used assay evaluates cell viability by measuring the metabolic activity of cells. In this study, melanoma cells were exposed to various concentrations of the alginate films. The findings shed light on the remarkable performance of alginate-based films loaded with PEG-coated gold nanoparticles in various concentration ranges (15 to 500 micrograms/ml). In fact, the observed survival rates consistently hovered around 100%. These notable results serve as robust evidence, reassuring us about the impressive safety and compatibility of this combined formulation. The high survival rates suggest that the interaction between the alginate-based films and PEG-coated gold nanoparticles avoids any adverse effects and attests to their harmonious coexistence. These findings not only underscore the potential of this combination but also encourage further exploration of its applications and benefits.

## Conclusion

With the advancement of nanotechnology, various nanoparticles with different structures, shapes, and compositions have shown promising potential in tissue engineering, hydrogel fabrication, therapeutic coatings, cancer diagnosis, and treatment, among other applications. Compared to other nanoparticles, gold nanoparticles (AuNPs) have demonstrated numerous advantages in these fields. In the course of this project, a composite hydrogel containing functionalized AuNPs was developed as a novel biomaterial. The study aimed to investigate the properties and characteristics of gold nanoparticles with a biocompatible polymer coating, polyethylene glycol (PEG). Considering the complex nature of the biomaterial, the process was divided into three stages: synthesis of AuNPs, functionalization of AuNPs, and film preparation. Each stage was successfully performed and confirmed using various characterization techniques. The results of the toxicity evaluation with MTT assay provided insights into the biocompatibility and safety of the alginate-based films loaded with PEG-coated gold nanoparticles. The fabrication of this hybrid hydrogel proved to be a robust and highly repeatable method. The presence of polyethylene glycol allowed for the loading of various drugs on the surface of the gold nanoparticles and, consequently, within the hydrogel, making it a suitable candidate for potential applications in tissue engineering.

## Authors’ Contributions

FS A performed experiments and analysis and interpretation of results. N HN provided supervision and funding acquisition, and helped revise and edit the article. A H, NA, and MG helped prepare the draft manuscript. N A supervised, helped acquire funds, conceived the study and design, analyzed and interpreted the results, and performed visualization.

## Conflicts of Interest

The authors declare that no conflict of interest exists.
